# Identifying Hemophagocytic Lymphohistiocytosis and Describing Outcomes Using Computable Phenotypes: Retrospective Cohort Study

**DOI:** 10.2196/87347

**Published:** 2026-03-26

**Authors:** Suheyla Ocak, Martin Yi, Agata Wolochacz, Ida Mehrdadi, Ahmed Naqvi, Sumit Gupta, Lillian Sung, Adam P Yan

**Affiliations:** 1 Division of Haematology/Oncology, Hospital for Sick Children, 555 University Avenue, Toronto, ON, M5G 1X8, Canada, 1 4168131500; 2 Department of Pediatric Hematology and Oncology, Cerrahpasa Medical Faculty, Istanbul University-Cerrahpasa, Istanbul, Turkey; 3 Child Health Evaluative Sciences, The Hospital for Sick Children, Toronto, ON, Canada

**Keywords:** HLH, electronic health record, pediatrics, computable phenotype, oncology, hemophagocytic lymphohistiocytosis

## Abstract

**Background:**

Hemophagocytic lymphohistiocytosis (HLH) is a life-threatening hyperinflammatory syndrome that requires rapid diagnosis and intervention. However, identifying these patients is difficult because the HLH-2004 diagnostic criteria are complex and not always captured systematically in electronic health records (EHRs). Furthermore, it is unclear how clinicians use these criteria to diagnose HLH and make treatment decisions. There is a critical need for validated computable phenotypes to accurately identify patients and study treatment-related outcomes in HLH.

**Objective:**

The aim of this study is to compare different approaches to using the EHR to build computable phenotypes of patients with HLH and to evaluate characteristics and outcomes of patients meeting the HLH-2004 diagnostic criteria who received HLH-directed therapies compared to those who did not.

**Methods:**

Three approaches to computable phenotype development in the EHR were taken by identifying patients (1) with an HLH-specific *International Statistical Classification of Diseases, Tenth Revision* (*ICD-10*) code, (2) with an HLH-specific treatment plan, and (3) meeting the HLH-2004 clinical criteria for diagnosis of HLH. Among patients who met the HLH-2004 criteria, we evaluated the characteristics and outcomes of patients who received HLH-directed therapies compared to those who did not. HLH treatment was defined as either any chemotherapy or HLH-specific therapy (dexamethasone, methylprednisolone, anakinra, ruxolitinib, cyclosporine, etoposide, or emapalumab).

**Results:**

We identified 388 patients with possible HLH across the three cohorts. An HLH *ICD-10* diagnosis (n=220) and meeting 5 or more clinical criteria (n=245) were much more common than a HLH treatment plan (n=42). Among the patients meeting HLH-2004 clinical criteria, 193 (79%) received HLH-directed therapy. There was no difference in any specific HLH criteria between those who did and did not receive HLH-directed therapy. In-hospital mortality was very high among both groups and was 15% among those who received HLH-directed therapy and 13.5% among those who did not receive HLH-directed therapy. Among 1325 patients with an elevated ferritin and fever, only 252 (19%) met >5 clinical criteria.

**Conclusions:**

Constructing HLH cohorts from EHR data is challenging, with diagnosis codes, treatment plans, and clinical criteria each capturing distinct but overlapping populations.

## Introduction

Hemophagocytic lymphohistiocytosis (HLH) is a rare but often life-threatening syndrome represented by excessive inflammation resulting in organ dysfunction and potentially death [1]. Early recognition and treatment of HLH are critical to patient outcomes. Traditionally, HLH has been classified as primary, where there is a documented or presumed genetic etiology [2,3], or secondary, where excessive inflammation can be attributed to a trigger such as infection, malignancy, or a rheumatological condition in the absence of a genetic etiology [4]. Although there are an increasing number of genes identified responsible for primary HLH [5], there are patients with presumed primary HLH without a known mutation. Further, patients with both primary and secondary HLH may require HLH-directed therapy. Consequently, diagnostic criteria have been developed for HLH (Multimedia Appendix 1), which often guide decision-making about treatment initiation. Criteria were initially proposed in 2004 [2,3] and recently updated in 2024 [3].

In general, diagnosis of HLH can be made based upon molecular or functional cellular findings consistent with HLH in combination with meeting at least 5 clinical criteria (Textbox 1). While these criteria have been widely used for diagnosis and treatment decision-making, there have been questions raised about the specificity of these criteria, particularly as they relate to secondary HLH [4]. HLH frequently presents with nonspecific clinical features that overlap with severe infection, sepsis, malignancy, and other inflammatory syndromes, leading to substantial diagnostic uncertainty in real-world clinical practice. Most studies of HLH have focused on small cohorts of participants who have received a clinical diagnosis of HLH. To evaluate the utility of HLH criteria, it may be informative to determine the association of each individual HLH criterion against an overall clinical HLH diagnosis, whether HLH-specific treatments received or an HLH outcome such as mortality.

Textbox 1. **HLH-2004 and HLH-2024 diagnostic criteria.**

**HLH-2004** [3]

Criterion 1 or 2 is fulfilled.

1. A molecular diagnosis consistent with hemophagocytic lymphohistiocytosis (HLH)
2. Diagnostic criteria for HLH fulfilled (5 of the 8 criteria below):

- Fever
- Splenomegaly
- Cytopenias (affecting ≥2 of 3 lineages in the peripheral blood)
  - Hemoglobin <90 g/L (hemoglobin <100 g/L in infants <4 wk)
  - Platelets <100×10^9^/L
  - Neutrophils <1.0×10^9^/L
- Hypertriglyceridemia and/or hypoﬁbrinogenemia
  - Fasting triglycerides ≥3.0 mmol/L (ie, ≥265 mg/dL)
  - Fibrinogen ≤1.5 g/L
- Hemophagocytosis in bone marrow or spleen or lymph nodes. No evidence of malignancy.
- Low or no natural killer cell activity (according to local laboratory reference)
- Ferritin ≥500 µg/L
- CD25 (ie, soluble IL-2 receptor) ≥2400 U/mL

**HLH-2024** [2]

Criterion 1, 2, or 3 below is fulfilled.

1. Functional cellular findings consistent with familial HLH (FHL) in a patient with signs/symptoms suggestive of HLH
2. A molecular diagnosis consistent with FHL in a patient with signs/symptoms suggestive of HLH
3. Clinical diagnostic criteria for FHL with at least 5 of the 7 criteria below fulfilled:

- Fever ≥38.5°C
- Splenomegaly (≥2 cm below the costal margin)
- Cytopenias (affecting ≥2 of 3 lineages in the peripheral blood)
  - Hemoglobin <90 g/L (hemoglobin <100 g/L in infants <4 wk)
  - Platelets <100×10^9^/L
  - Neutrophils <1.0×10^9^/L
- Hypertriglyceridemia and/or hypoﬁbrinogenemia
  - Fasting triglycerides ≥3.0 mmol/L
  - Fibrinogen ≤1.5 g/L
- Hemophagocytosis
- Ferritin ≥500 µg/L
- sCD25 (ie, soluble IL-2 receptor) ≥2400 U/mL

Most studies of patients with HLH use small, highly curated datasets. Such studies are therefore limited in their ability to evaluate how well diagnostic criteria perform in broader, unselected clinical populations. Few studies have leveraged electronic health record (EHR) data, which may represent broader populations and contain a wider range of clinical information. Leveraging EHR data to facilitate clinical research of HLH requires the construction of an accurate patient cohort. Given that EHR data are not entered with the goal of facilitating future downstream research, manually entered data such as diagnosis codes can often be incorrect or missing. Computable phenotypes are machine-evaluable definitions for a given condition developed using EHR data. Multisource computable phenotypes incorporating laboratory results, medications, procedures, and clinical events may improve case ascertainment and reduce bias compared with diagnosis codes alone [6]. To our knowledge, no attempt has been made to use diverse EHR data to develop a machine-evaluable approach to HLH identification [7-9]. It is unknown how alternative EHR-based definitions perform for a clinically heterogeneous and dynamically evolving syndrome such as HLH.

The primary objective of this study was to compare various approaches to using EHR data to build a computable phenotype of patients with HLH—specifically, we compared three approaches: (1) HLH-specific diagnosis code usage, (2) HLH-specific treatment plan application, and (3) HLH clinical criteria, among all patients at a pediatric hospital. Our hypothesis was that the use of diagnosis codes and treatment plans would undercapture patients and the use of clinical criteria would overcapture patients. Among patients who met at least five HLH clinical criteria, the secondary objective was to explore characteristics and outcomes among those who received HLH-directed therapy versus those who did not.

## Methods

### Ethical Considerations

This observational study was approved by the Research Ethics Board at The Hospital for Sick Children (SickKids). The requirement for informed consent and assent were waived given the retrospective nature of the study. In keeping with our Research Ethics Board approval, no potentially identifying information of any participant is disclosed in this report. All data are presented as aggregates.

### Data Source

The data source was the SickKids Enterprise-wide Data in Azure Repository (SEDAR) [10]. SEDAR is a curated and validated version of the Epic Clarity database that lives in Microsoft Azure. To create SEDAR, we transformed Clarity first into a Filtered Schema that retains only valid SickKids patients, then into a curated schema where the data are structured into clinically relevant units such as patients, encounters, laboratory tests, and medication administrations. During SEDAR creation, all data were validated via random sampling. This project focused on the following SEDAR tables: patient, diagnoses, cancer treatment plans, hospital encounter, nonhospital encounter, medication administration, prescriptions, laboratory tests, pathology results, flowsheets, and notes.

### Operationalizing HLH Criteria

We used the HLH-2004 criteria (as opposed to the HLH-2024 criteria) as these would have been the criteria in place for most of the patient cohort. The time window to evaluate clinical criteria was centered on an episode, which was usually an inpatient admission spanning admission to discharge. A criterion was considered met if it occurred at any point during the episode. Five of the criteria were based on the laboratory results table (ferritin, cytopenia, hypertriglyceridemia or hypofibrinogenemia, sCD25, and low natural killer cell activity) and were considered met if the patient ever had a value above or below the criteria, as appropriate. Fever was defined as an oral temperature of at least 38.3°C once or 38.0°C to 38.2°C for at least one hour [11]. If a laboratory test was not performed or a criterion was not met, the criteria was categorized as not abnormal.

Two of the criteria required searching of text. Hemophagocytosis was identified by searching for “h(a)emophagocytosis” or “h(a)emophagocytic” in all pathology results. These reports were manually reviewed to identify true hemophagocytosis. Splenomegaly was identified by searching all notes for any of the following terms “splenomegaly,” “big spleen,” “organomegaly,” and “enlarged spleen.” Negative terms were excluded using the following terms within 3 words previous to the splenomegaly term: “no,” “none,” “absence,” “without,” and “negative.” The number of mentions were too high to manually review each note. Thus, a random sample of 20 notes underwent chart review to validate the approach. We found that 19/20 were correct. One of 20 was incorrect. We considered this satisfactory to proceed.

### Eligibility Criteria

We established three cohorts of HLH “diagnosis” based upon encounters that occurred between June 2, 2018, and May 31, 2025. First, patients with a coded diagnosis of HLH were those with an *International Statistical Classification of Diseases, Tenth Revision* (*ICD-10*) code of D76.1 (hemophagocytic lymphohistiocytosis) or D76.2 (hemophagocytic syndrome, infection-associated). Second, cancer treatment plans are electronic care plans that are a component of Epic’s Beacon oncology module. All chemotherapy at our institution must be ordered within a treatment plan; however, treatment plan use is restricted to use by oncologists. To that end, an oncologist would therefore order a drug such as emapalumab within a treatment plan, while a rheumatologist using the same drug would not. We manually identified either treatment plan or protocol display names that included “HLH” or “h(a)emophagocytosis.” Use of a treatment plan was defined as having an HLH-specific treatment plan applied in Epic.

The third approach consisted of identifying the number of HLH-2004 clinical criteria within an encounter. The HLH-specific encounter was the encounter with the maximum number of criteria. If there was more than one encounter with this number of criteria, the first encounter was selected. For establishment of HLH based on clinical criteria, we identified encounters with at least 5 criteria within that encounter regardless of encounter length.

Given that there is no gold-standard adjudicated HLH cohort, determining which computable phenotype for cohort creation was most accurate was not possible with this data set.

### Procedure

We were interested in two measures of whether patients received HLH treatment; these might be administered within or outside of an HLH treatment plan. First, we considered any chemotherapy. Second, we considered HLH-directed therapy, which we defined as receipt of any dosage of dexamethasone, methylprednisolone, anakinra, ruxolitinib, cyclosporine, etoposide, or emapalumab [12-16].

We compared demographic features, HLH-related variables, and clinical outcomes among those with at least 5 HLH clinical criteria for those who received HLH-directed therapy (yes vs no) during the encounter where the criteria were met. The goal of this analysis was description and hypothesis generation given the possibility of confounding by indication for treatment. Demographic features were sex and age group (0-<1, 1‐4, 5‐14, and ≥15 years). HLH-related variables were whether there was an *ICD-10* HLH diagnosis associated with that encounter, timing of *ICD-10* HLH diagnosis relative to the encounter, and specific clinical criteria met. Clinical outcomes were services involved, length of stay, intensive care unit admission, in-hospital mortality, 30-day mortality, and whether the patient underwent stem cell transplantation following the encounter. To calculate 30-day mortality, we identified all patients with a date of death within 30 days of the visit where they met the HLH-2004 criteria. We also evaluated the 5 most common admission diagnoses among the cohort with at least 5 clinical criteria. Primary admission diagnosis was derived from the admitting diagnosis field in our EHR.

As an exploratory objective, we hypothesized that high ferritin and fever are common conditions in pediatric patients. Thus, we identified all encounters that met the HLH-2004 ferritin and fever criteria. Any encounter where the patient met both criteria at any point during the encounter were counted. We then described the distribution of the other HLH-2004 criteria in this cohort and stratified encounters by whether there were at least 5 clinical criteria present during an HLH-specific encounter.

### Analysis

To compare patients who received HLH-directed therapy (yes versus no) among patients who met at least 5 clinical criteria, we used the Wilcoxon rank-sum test for continuous variables and *χ*^2^ or Fisher exact test for categorical variables. Absolute risk differences in in-hospital mortality, 30-day mortality, and median length of stay were calculated with 95% confidence intervals. Analyses were performed with R (version 4.2.2; R Foundation for Statistical Computing).

## Results

**Figure 1.**
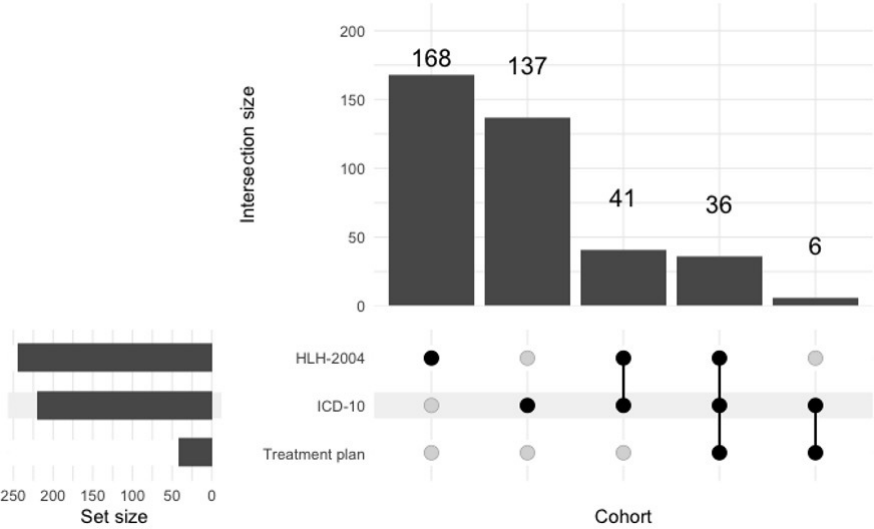
Intersection of HLH cohorts derived using three different approaches to cohort creation (N=388 patients). HLH: hemophagocytic lymphohistiocytosis; *ICD-10*: *International Statistical Classification of Diseases, Tenth Revision*.

Figure 1 shows the intersection of the 388 patients identified as having HLH using our three approaches to HLH-cohort creation using three different computable phenotypes (diagnosis code, treatment plan, or HLH criteria). All patients with an HLH-specific treatment plan also had either an *ICD-10* HLH diagnosis or met clinical HLH criteria. Only 36 patients were identified by all three methods as having HLH. Table 1 shows the number of patients with an HLH *ICD-10* diagnosis code, an HLH treatment plan, or who met at least 5 HLH clinical criteria. An HLH *ICD-10* diagnosis (n=220 patients) and meeting 5 or more clinical criteria (n=245) were much more common than an HLH treatment plan (n=42 patients). Among the 220 patients with an HLH *ICD-10* code, only 35 patients received chemotherapy during the HLH-specific encounter. However, 85 patients received HLH-directed therapy during the HLH-specific encounter. Conversely, among the 245 patients who met at least 5 clinical criteria, 78 patients received chemotherapy and 193 patients received HLH-directed therapy during the HLH-specific encounter.

**Table 1.** Number of patients with possible hemophagocytic lymphohistiocytosis (HLH) by different approaches.

	HLH *ICD-10*^a^ diagnosis code	HLH treatment plan	Five or more HLH criteria
Total number of patients	220	42	245
Number of patients with chemotherapy during HLH-specific encounter^b^	35	37	78
Number of patients with chemotherapy during any encounter	73	38	99
Number of patients with chemotherapy or HLH-directed therapy^c^ during HLH-specific encounter	85	41	198
Number of patients with chemotherapy or HLH-directed therapy during any encounter	143	42	213
Number of patients with HLH-directed therapy during HLH-specific encounter	85	41	193
Number of patients with HLH-directed therapy during any encounter	142	42	212

a *ICD-10*: *International Statistical Classification of Diseases, Tenth Revision.*

b HLH-specific encounter is associated with first HLH diagnosis, first HLH treatment plan, or first encounter with the maximum number of criteria met.

c HLH-directed therapy includes dexamethasone, methylprednisolone, anakinra, ruxolitinib, cyclosporine, etoposide, and emapalumab.

Table 2 shows the demographic features of unique patients who met 5 or more HLH criteria stratified by receipt of HLH-directed therapy versus not. The median age of the cohort was 7.7 years (IQR 1.4‐13.9). Of the 193 patients who received HLH-directed therapies, 67.9% (n=131) only received steroids and 29% (n=56) received steroids in combination with an additional HLH-directed therapy. Those with sepsis were less likely to receive HLH-directed therapy (13.5% vs 4.7%; *P*=.05). Those patients receiving HLH-directed therapy were more likely to have an *ICD-10* HLH diagnosis code during that encounter (*P*=.001). Notably, there was no difference in any specific HLH criteria between those who did and did not receive HLH-directed therapy although high ferritin was almost universal in both groups. The absolute difference in in-hospital mortality and 30-day mortality rates between the groups was 1.5% (95% CI –1.1 to 1.1) and 1.2% (95% CI –1.2 to 1.1), respectively. The absolute difference in median length of stay between the two groups was 12.6 days (95% CI 4.1 to 22.4).

**Table 2.** Number of patients with at least 5 hemophagocytic lymphohistiocytosis (HLH) criteria stratified by receipt of HLH-directed therapy.

	HLH-directed therapy (n=193)	No HLH-directed therapy (n=52)	*P* value
Demographic features			
Male sex, n (%)	96 (49.7)	27 (51.9)	.90
Age group in years, n (%)			.34
0-<1	43 (22.3)	13 (25)	
1‐4	44 (22.8)	12 (23.1)	
5‐14	65 (33.7)	14 (26.9)	
≥15	41 (21.2)	12 (23.1)	
Primary admission diagnosis^a^, n (%)			
Fever	37 (19.2)	14 (26.9)	.30
Sepsis	9 (4.7)	7 (13.5)	.05
Leukemia	12 (6.2)	2 (3.8)	.75
Acute lymphoblastic leukemia	8 (4.1)	4 (7.7)	.49
Neutropenia	6 (3.1)	3 (5.8)	.62
HLH-related variables			
*ICD-10*^b^ HLH diagnosis, n (%)	77 (31.4)	5 (9.6)	<.001
*ICD-10* HLH diagnosis relative to encounter, n (%)			.001
Before	8 (4.1)	0 (0)	
During	55 (28.5)	3 (5.8)	
After	9 (4.7)	2 (3.8)	
Never had HLH diagnosis	121 (62.7)	47 (90.4)	
HLH criteria present, n (%)			
High ferritin	190 (98.4)	52 (100)	.85
Fever	177 (91.7)	47 (90.4)	.98
Cytopenia of at least 2 lineages	176 (91.2)	48 (92.3)	>.99
Hypertriglyceridemia or hypofibrinogenemia	183 (94.8)	47 (90.4)	.39
Splenomegaly	175 (90.7)	46 (88.5)	.83
sCD25^c^	91 (47.2)	26 (50)	.84
Hemophagocytosis	33 (17.1)	3 (5.8)	.07
Low natural killer cell activity^c^	13 (6.7)	1 (1.9)	.32
Clinical outcomes			
Services involved, n (%)			
Oncology	109 (56.5)	22 (42.3)	.01
Hematology	103 (53.4)	33 (63.5)	.25
Rheumatology	89 (46.1)	16 (30.8)	.07
Infectious diseases	159 (82.4)	45 (86.5)	.62
Immunology	46 (23.8)	13 (25)	>.99
Neurology	69 (35.8)	17 (32.7)	.80
Median length of stay (IQR)	33.4 (81.1-14.3)	20.8 (33-8.5)	.001
Intensive care unit, n (%)	111 (57.5)	22 (42.3)	.07
In-hospital mortality, n (%)	29 (15)	7 (13.5)	.95
Mortality within 30 days, n (%)	32 (16.6)	8 (15.4)	>.99
Stem cell transplant following encounter, n (%)	13 (6.7)	1 (1.9)	.32

a Five most common admitting diagnoses for patients with at least 5/8 HLH criteria (n=245).

b *ICD-10*: *International Statistical Classification of Diseases, Tenth Revision*.

c Number of encounters where measured for HLH-directed therapy (yes versus no): sCD25: 135 versus 36; natural killer cell activity: 53 versus 7.

Table 3 shows that among the 1325 encounters (where individual patients can have more than one encounter) with high ferritin and fever, cytopenia, hypertriglyceridemia or hypofibrinogenemia, and splenomegaly were very common. Of the 1325 encounters, 19% (n=252) had >5 HLH criteria. Even among those with less than 5 clinical criteria, cytopenia was present in 44% (n=475) of encounters while hypertriglyceridemia or hypofibrinogenemia and splenomegaly were present in 24% (n=255) and 27% (n=290) of encounters, respectively.

**Table 3.** Distribution of criteria among encounters with high ferritin and fever^a^.

	All encounters (N=1325 encounters), n (%)	5 or more criteria (n=252 encounters), n (%)	<5 criteria (n=1073 encounters), n (%)
Cytopenia of at least 2 lineages	706 (53)	231 (92)	475 (44)
Hypertriglyceridemia or hypofibrinogenemia	485 (37)	230 (91)	255 (24)
Splenomegaly	519 (39)	229 (91)	290 (27)
sCD25^b^	159 (12)	114 (45)	45 (4)
Hemophagocytosis	27 (2)	27 (11)	0 (0)
Low natural killer cell activity^b^	12 (1)	11 (4)	1 (0.1)

a The table represents all encounters where ferritin and fever met the threshold for HLH diagnosis without considering if the patient was diagnosed with HLH by any approach. There may be multiple episodes per patient.

b Number of encounters where measured for ≥5 versus <5 criteria were as follows: sCD25: 172 (68%) versus 165 (15%); natural killer cell activity: 55 (22%) versus 24 (2%).

Multimedia Appendix 1 shows the distribution of clinical criteria among those with an HLH *ICD-10* code and among those with at least 5 clinical criteria. Some patients with their first HLH *ICD-10* diagnosis code had no clinical criteria.

## Discussion

In this study, we compared three approaches to constructing an EHR-derived cohort of pediatric patients with HLH: (1) *ICD-10* diagnosis codes, (2) HLH-specific treatment plans, and (3) fulfillment of HLH-2004 clinical criteria. Our findings demonstrate substantial variation in the populations identified by each approach, with diagnosis codes and clinical criteria identifying a far greater number of encounters than HLH-specific treatment plans. We also identified that among patients meeting at least five HLH-2004 clinical criteria, a large proportion received HLH-directed therapy, and that among a cohort of patients with fever and an elevated ferritin, a substantial portion have other findings of HLH such as cytopenia, hypertriglyceridemia or hypofibrinogenemia or splenomegaly.

Comparing our three approaches to EHR-based cohort construction yields numerous interesting insights. First, we observed that HLH *ICD-10* codes were relatively common in our dataset. A large proportion of patients with an HLH *ICD-10* code did not receive HLH-directed therapy, suggesting that coded diagnoses may be used for suspected but unconfirmed HLH. Conversely, while HLH treatment plans were highly specific, they were rarely used. This is likely because many pediatric patients with HLH do not require chemotherapy and receive HLH-directed medications that are ordered outside of a chemotherapy plan. Thus, the use of treatment plans, which are likely the most accurate way to identify HLH-directed medications, likely underestimates the true HLH population. Clinical criteria, while nonspecific, identified the broadest cohort, consistent with the recognition that HLH represents a clinical syndrome overlapping with other hyperinflammatory states. The exclusive use of these criteria can also be problematic in an EHR cohort, where certain laboratory tests may be done at a referring institution such as for a patient with HLH being referred for a hematopoietic stem cell transplant. This is supported by the fact that we had some patients who met zero HLH clinical criteria but had an HLH *ICD-10* code applied. Future research leveraging EHR data to assemble an HLH cohort needs to consider the advantages and disadvantages of different techniques for cohort assembly to select the best approach for a given project. For example, a researcher interested in understanding the diagnostic odyssey of patients with HLH prior to treatment to improve time to diagnosis might use diagnostic criteria as this would more broadly capture patients compared to using a more narrow approach like diagnostic codes. Future work should also explore the usage of a combination of approaches such as having both HLH-criteria met and an HLH diagnosis code and using additional filters in the treatment approach such as dosages or duration for medications that might be used for indications other than HLH management such as dexamethasone. It will also be important to validate these approaches at other institutions to understand generalizability.

Using our newly developed EHR-based cohort of children with HLH, we were able to generate new insights into the management and outcomes of patients with HLH. Of the 245 patients who met the HLH-2004 criteria for HLH, 27% (n=52) received no HLH-directed therapy. No differences were identified in the occurrence of HLH-specific criteria such as ferritin or fever between the two cohorts. From this work, it remains unclear as to what drove the difference in clinical decision-making to start HLH-directed therapy for patients meeting 5 criteria. We did identify that patients who did not receive HLH-directed therapy were more likely to have sepsis than those who did [17]. This is not surprising given that we know that sepsis and HLH have an overlapping presentation. Patients who received HLH-directed therapy also had a significantly longer length of stay (33.4 vs 20.8 d). It is important to note that regardless of whether or not a patient who met 5 criteria received therapy, their 30-day mortality risk was approximately 1 in 6. This is similar to other contemporary studies of patients treated for HLH, where mortality rates have ranged from 23% to 55% [14,18-20]. Given the high mortality rate in those who did not receive therapy, it raises the question of whether or not we are failing to treat a subset of patients with HLH with appropriate HLH-directed therapies.

We also evaluated the distribution of HLH criteria within an EHR-derived cohort of patients with high ferritin and fever. An elevated ferritin and fever are often used as the entrance criteria into a “rule-out” HLH algorithm at many institutions that results in the ordering of additional HLH-specific labs or consultation of an HLH-specific team such as hematology/oncology and/or rheumatology [16,21]. In this cohort that mirrors a potential cohort of patients where an HLH work-up and diagnosis may be considered, we identified that a significant proportion of patients will meet additional HLH criteria. However, only 20% (n=252) of the patients in this cohort met >5 HLH criteria, suggesting that if fever and ferritin are used as entrance criteria into an HLH algorithm, 5 patients would need to be evaluated to identify 1 patient meeting the HLH clinical criteria.

The strengths of this study are access to all data in a curated and validated EHR data set, which allows for the inclusion of patients without an HLH diagnosis. Unlike prior studies restricted to curated clinical cohorts, our approach provides insight into the challenges of applying HLH diagnostic frameworks in real-world, unselected populations. However, limitations of this study include that it was done at a single pediatric institution, so results may not generalize to other institutions. Specifically, in the Canadian context, we may underutilize ICD codes compared to American peers given different implications on billing and remuneration [22]. Second, we were limited in validating the performance of our cohorts against a gold-standard cohort, as no such curated dataset exists that includes both primary and secondary patients with HLH. Third, EHR-derived data are prone to misclassification. Diagnosis codes may not reflect confirmed diagnoses, laboratory results may be incomplete (as SickKids is a large referral center so some labs may have been done at a referring institution and may not have been included in our dataset), and text-mining strategies (eg, for splenomegaly) have imperfect specificity. Fourth, we centered our analysis on the HLH-2004 criteria given these were the criteria being used by clinicians during our study period. The HLH-2024 criteria have since been published, which differ slightly from the HLH-2004 criteria by removal of the natural killer cell activity criterion. Given that only 5% (n=60) of patients had natural killer cell activity testing performed in our cohort, removal of this criteria is unlikely to impact results. Finally, treatment receipt was defined by medication administration records, which may not fully capture the intent of medication prescribing, and this analysis may be confounded by medication indication.

Constructing HLH cohorts from EHR data is challenging, with diagnosis codes, treatment plans, and clinical criteria each capturing distinct but overlapping populations. While clinical criteria identify the broadest group, they lack specificity, underscoring the limitations of applying rigid diagnostic frameworks to real-world EHR data. Developing accurate, machine-evaluable HLH phenotypes will be critical to advancing both clinical care and research in this rare but important condition.

## Supplementary material

10.2196/87347 Multimedia Appendix 1 Distribution of HLH clinical criteria.

## Abbreviations

EHR: electronic health record
HLH: hemophagocytic lymphohistiocytosis
*ICD-10*: *International Statistical Classification of Diseases, Tenth Revision*
SEDAR: SickKids Enterprise-wide Data in Azure Repository
SickKids: The Hospital for Sick Children
