# Supplementary material for: Identifying Hemophagocytic Lymphohistiocytosis and Describing Outcomes Using Computable Phenotypes: Retrospective Cohort Study
Source: JMIR Cancer. 2026 Mar 26;12:e87347. doi: 10.2196/87347 (PMC13021101; doi:10.2196/87347)
Supplement: Multimedia Appendix 1 [file cancer-v12-e87347-s001.docx]

**Appendix 1: Distribution of Number of Criteria Met for those with HLH Diagnosis and those with At least Five Criteria**

| **Number of Criteria** | **HLH ICD10 Diagnosis (N=220)** | **At least 5 Criteria (N=243)** |
| --- | --- | --- |
| 0 | 33 |  |
| 1 | 27 |  |
| 2 | 22 |  |
| 3 | 18 |  |
| 4 | 43 |  |
| 5 | 38 | 171 |
| 6 | 31 | 62 |
| 7 | 8 | 10 |
| 8 | 0 | 0 |
